# A UAV‐based portable health clinic system for coronavirus hotspot areas

**DOI:** 10.1049/htl2.12035

**Published:** 2022-09-28

**Authors:** Mustafa Siham Qassab, Qutaiba Ibrahim Ali

**Affiliations:** ^1^ Computer Engineering Department University of Mosul Mosul Iraq

**Keywords:** COVID‐19, drones, full curfew, portable health clinic, remote healthcare system

## Abstract

This study applied the World Health Organization (WHO) guidelines to redesign the Portable Health Clinic (PHC), as a Remote Healthcare System (RHS), for the spread of COVID‐19 containment. Additionally, the proposed drone‐based system not only collects people data but also classifies the case according to the main symptoms of coronavirus using the COVID‐19 triage process (CT‐process) based on the analysis of measurement readings taken from patients, where drones are used in a swarm as a PHC platform and are equipped with the required sensors and essential COVID‐19 medications for testing and treating people at their doorstep autonomously when a full curfew is imposed. This paper describes a complete framework and proposes currently in production hardware to build the suggested system, considering the effect of the extra payload weight on drone's durability. In addition, part of the proposed application was simulated using OPNET simulation tool. This work highlights the main aspects that should be considered when designing drone swarm‐based system and distributing the roles on system nodes with the main focus on the controlling messages for inter‐swarm and intra‐swarm communication and coordination.

## 1 INTRODUCTION

Coronavirus disease, or COVID‐19, is an infectious illness that has been discovered recently [1]. This virus was unknown before the Wuhan chain occurrence in December 2019, and by April 2022 over 490 million people globally are infected and over 6 million have died [2]. COVID‐19′s most like to appear symptoms are fever, fatigue, and dry cough. Furthermore, several patients also experienced runny noses, nasal clog, sore throat, throbbing pain, or diarrhoea [3]. Individuals get infected by this virus through tiny beads when coughing, sniffling, or talking during close contact [4], these beads fall to a surface or ground making contaminations over long distances with a survival time of up to 72 h. On 11 March 2020, the WHO announced it was a pandemic. At that point, numerous countries have imposed nationwide lockdown, especially in COVID‐19 hotspots with an aggressive promotion of the social distancing concept in the media to raise awareness. A hotspot is a district where reports indicate a relatively larger number of confirmed COVID‐19 infections [5]. This motivates the idea of this paper, where populations under a curfew are being tested for COVID‐19 symptoms at their doorsteps using a swarm of robots with minimal human intervention. The uniqueness of the proposed approach is that the PHC platform is fully automated and delivers the COVID‐19 testing and medication services to one's doorstep during a full curfew.

The rest of the paper is organized as follows; Section 2 compares traditional and drone‐based COVID‐19 data collection, and the design methodology is explained in Section 3. The redesigned PHC system is discussed in Section 4, then the discussion and comparison with other works are mentioned in Section 5. Finally, conclusions are brought in Section 6.

## 2 COVID‐19 DATA COLLECTION: TRADITIONAL VERSUS DRONE‐BASED METHODS

In [6], the authors mention a traditional COVID‐19 data collection approach named Vehicle‐Based Testing (VBT) used by the state Red Cross Organization, this approach could be briefly compared to our suggested drone‐based approach in Table 1.

**TABLE 1:** Traditional versus drone‐based COVID‐19 data collection approaches

Approach type		
Factor	Traditional approach	Drone‐based approach
Health workers are needed in field	Yes	No
There is a direct‐human contact when collecting data	Yes	No
Personal Protective Equipment (PPE) is needed	Yes	No
A mean for transportation is needed for health workers	Yes	No
Hygienic measures of staff are needed after completing the tour	Yes	No
Disinfecting the tools used in the tour	Yes	Yes
Parallel data collection possibility	No	Yes
Reduces the workload and stress on frontline healthcare workers	No	Yes
Reaching hard‐to‐access areas easily	No	Yes

## 3 DESIGN METHODOLOGY

WHO guidelines for combating COVID‐19 are followed here as a theoretical basis of the designed PHC system to meet the general requirements in the service, which include [7, 8, 9]:

‐ Primary screening and triage: Screening and isolation of all suspected COVID‐19 once in contact with the healthcare system to allow for proper prevention and control actions.
‐ Prevention and control: Isolation prevents viral transmission and quarantine may be at home or hospital depending on the patient's health status.
‐ Traceability and privacy: Contact tracing is essential to identify people who may have had exposure to a confirmed COVID‐19 patient and trace all possible contacts with keeping the patient's privacy.

### 3.1 The proposed platform specifications

Many researchers have adopted a commercial multi‐rotor UAV as a platform, which is the DJI

Phantom Pro v2, for different applications [10, 11, 12, 13] and hence it has been selected in this research. A network of autonomous drones creates a swarm consisting of one Leader Drone (LD) and many Slave Drones (SDs), specifications are illustrated in Table 2 [14]. It is also assumed that each drone has a Wi‐Fi module to enable network capability as in [15]. Additionally, a cellular communication technology, Worldwide inter‐operability for Microwave Access WiMAX in our case, is used by the LD for long communications, that is, with the base station. Therefore, the LD needs to be more powerful and durable than other SDs.

**TABLE 2:** Specification of DJI Phantom Pro v2 drone

**Aircraft and camera**	Weight: 1375 g, diagonal size (propellers excluded): 350 mm, max ascent speed: S‐mode: 6 m/s, P‐mode: 5 m/s, max descent speed: S‐mode: 4 m/s, P‐mode: 3 m/s, max speed: S‐mode: 45 mph (72 kph), A‐mode: 36 mph (58 kph), P‐mode: 31 mph (50 kph), max wind speed resistance: 10 m/s, max flight time: Approx. 30 min, satellite positioning systems: GPS/GLONASS, hover accuracy range (with GPS positioning): Vertical: ±0.5 m, Horizontal: ±1.5 m, camera sensor: 1‐inch CMOS, effective pixels: 20 M, max video bitrate: 100 Mbps, supported SD Card: microSD, capacity: 128 GB.
**Infrared sensing system**	Obstacle sensory range: 0.6–23 feet (0.2–7 m), FOV: 70° (Horizontal), ±10° (Vertical), measuring frequency: 10 Hz, operating environment: Surface with diffuse reflection material, and reflectivity > 8 percent (like wall, trees, humans etc.)
**Intelligent flight battery**	Capacity: 5870 mAh, voltage: 15.2 V, battery type: LiPo 4S, energy: 89.2 Wh, net weight: 468 g, charging temperature range: 41–104 °F (5–40°C), max charging power: 160 W

Furthermore, the payload means the extra hardware that is attached to or carried by the drone, and it depends heavily on the application that the UAV swarm will undertake. For the proposed system, the payload elements that will be adopted with their weights are given in Table 3.

**TABLE 3:** Drone's payload elements and their weights

**Payload element**	**Weight (g)**	**LD payload**	**SD payload**
Raspberry Pi 3 B [16]	42	Yes	Yes
Arducam OV5647 camera [17]	9	Yes	Yes
Dr. Prepare 20,000 mAh solar power bank [18]	451	Yes	Yes
MC34167TG switching voltage regulator [19]	2	Yes	Yes
Navio2 Autopilot flight controller [20]	23	Yes	Yes
SIM7600E HAT WiMAX adapter [21]	12.6	Yes	No
Dr. Prepare 10,000 mAh mini power bank [22]	201	Yes	No
Total weight	740.6	740.6	527

The Raspberry Pi is used as an onboard processing unit, the camera is an application‐specific need. There are two types of batteries mentioned, the 20,000 mAh is equipped in each drone as a power source for the Raspberry Pi kit and also to lengthen the drone's durability, especially it comes with a solar panel to charge the battery while in mission, and the other 10,000 mAh battery is only added to the LD because it needs to be more powerful as it is the hub of the swarm and represents the gateway to the BS. Additionally, the voltage regulator is needed so the drone can be also powered from the extra battery along with its original battery. Furthermore, the flight controller comes as a shield on the Raspberry Pi, or hat, and allows the Raspberry Pi to command the flight controller, and hence, control the drone. Finally, the WiMAX adapter is utilized to enable the LD to communicate with the BS at long distances.

### 3.2 The drones’ network architecture

**FIGURE 1:**
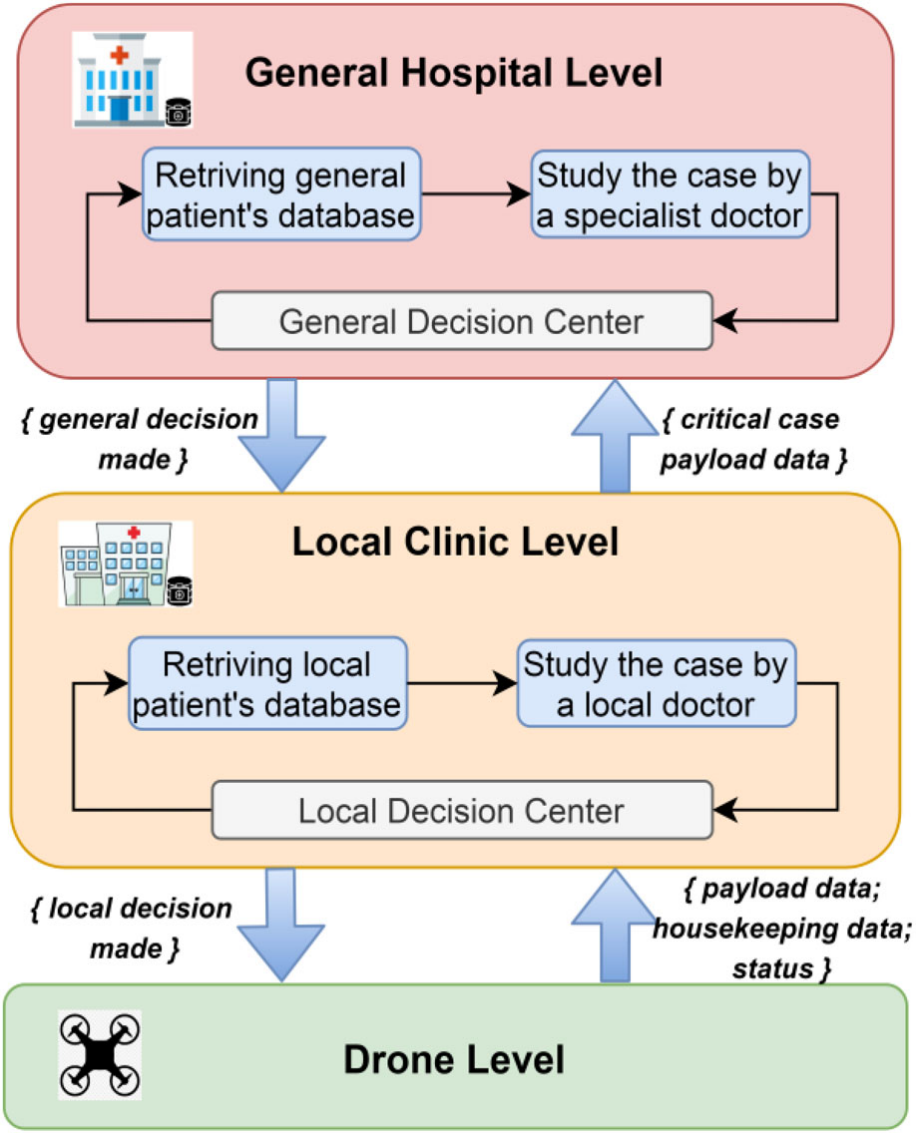
Overview of the system architecture

**FIGURE 2:**
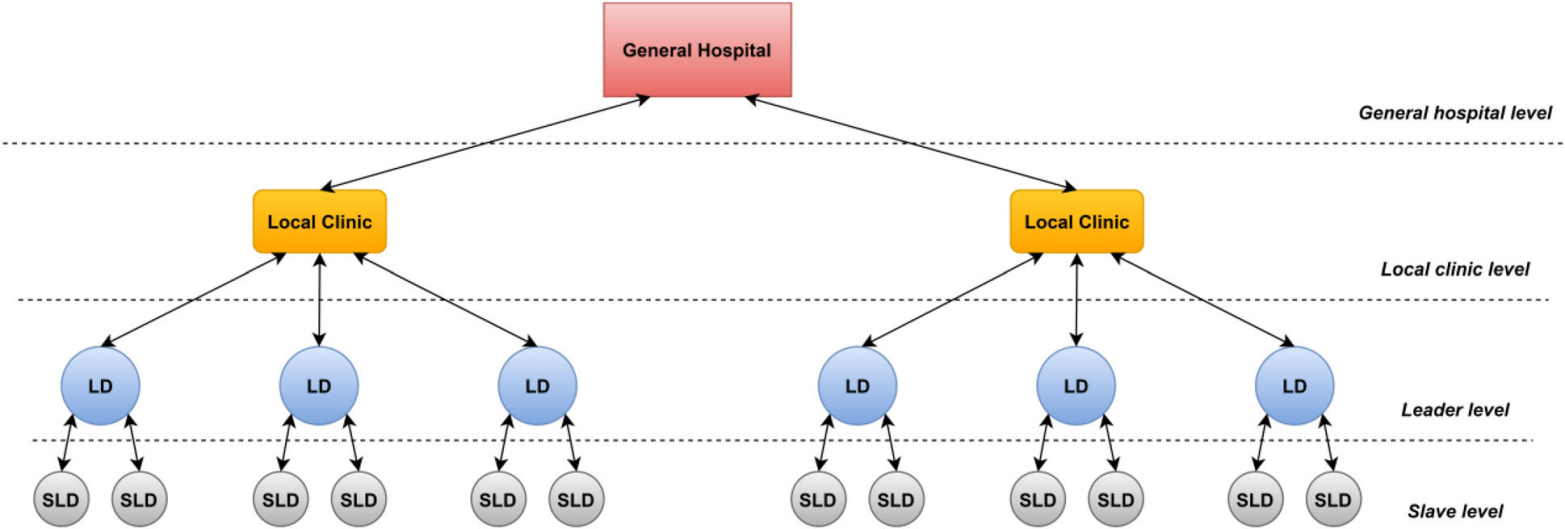
System layered architecture

The gathered data is initially processed at the drone level and then shared with upper‐level systems for further detailed processing and/or database storing. In our approach, we assume to have three operational levels or layers; drone, local clinic (or base station), and general hospital levels, Figure 1 shows an overview of the proposed system architecture, which makes it scalable in a hierarchical manner as shown in Figure 2. The drone level is comprised of SDs and one LD. The SDs collect COVID‐19 test data then process it and send a case status report, if the symptoms indicate that the case is infected, to the LD which acts as a swarm sink node and is connected to the local clinic level. At the local clinic level, a local clinic can run and manage multiple scanning instances in nearby areas simultaneously by deploying several drone swarms, and if any critical case is found then the general hospital is called for help. Therefore, a local clinic acts as a sink node for the drone swarms as it is connected to the general hospital. The general hospital level is the highest level, where it has the best available services for COVID‐19 containment in town including specialist doctors, medical equipment and supplies, and a general database for all city residents. Also, it supervises local clinics' operations and can intervene to diagnose emergency cases reported by local clinics upon their request.

### 3.3 Key SON functions

Improving multi‐UAV network performance could be realized by implementing Self‐organizing Network (SON) functions [23]. However, the decision on which SON functions to include in this paper was based on the design simplicity and low cost. Therefore, only essential and most relevant SON functions are selected and classified based on the phase of operation including:

#### 3.3.1 Self‐configuration SON functions

- Automatic generation of default parameters: For introducing a new host, different parameters should be assigned such as [24]: Network and security parameters, for example, Internet Protocol (IP) and server addresses and certificates. Software parameters like software version. Hardware parameters as firmware and required drivers. Radio network parameters as node parameters, neighbour relationships, transmission power etc.
- Network authentication: Mutual authentication of node and network is needed during the self‐configuration phase, especially when deploying new network elements [24].

#### 3.3.2 Self‐optimization SON functions

- Congestion control parameter optimization: It monitors network load, detects overload cases, measures the urgency degree of the overload conditions, and makes proper responses to get the system back to a feasible load situation in a controlled manner [24].
- Packet scheduling parameter optimization: Optimize resource efficiency by managing channel resource access while meeting Quality of Service (QoS) requirements [24].
- Reduction of energy consumption: Energy cost is one of high interest in the public, especially in drones. Therefore, energy efficiency must be considered [24].

#### 3.3.3 Self‐healing SON functions

- Cell outage prediction: It estimates which node is a candidate for an outage and provides information about the outage expected time, likelihood, scope, and type, then reports the cell outage detection function for further processing of possible causes [24].
- Cell outage detection: An outage should be detected within an adequately short time, for example, minutes, to respond effectively. The outage detection report may include [24]: The node ID for the malfunctioned node and the type and scope of the outage.
- Cell outage compensation: By autonomously adjusting network parameters to maximize performance and coverage, also fulfil mission requirements as much as possible [24].

### 3.4 Security concerns

The proposed drone‐based PHC system obtains residents’ COVID‐19 related data using drones and then transmits it to upper‐level health units. Data processing, security, and privacy concerns should be considered, so a secure tunnel, that uses end‐to‐end data encryption, can be utilized in collecting and transmitting data as only authenticated nodes can receive the data. Status reports are sent from the drone level to the local clinic level automatically; while a manual transmission of data is done between the general hospital and local clinics. All data is transmitted using an end‐to‐end secure data sharing tunnel.

## 4 REDESIGNED PHC SYSTEM FOR COMBATING COVID‐19

**FIGURE 3:**
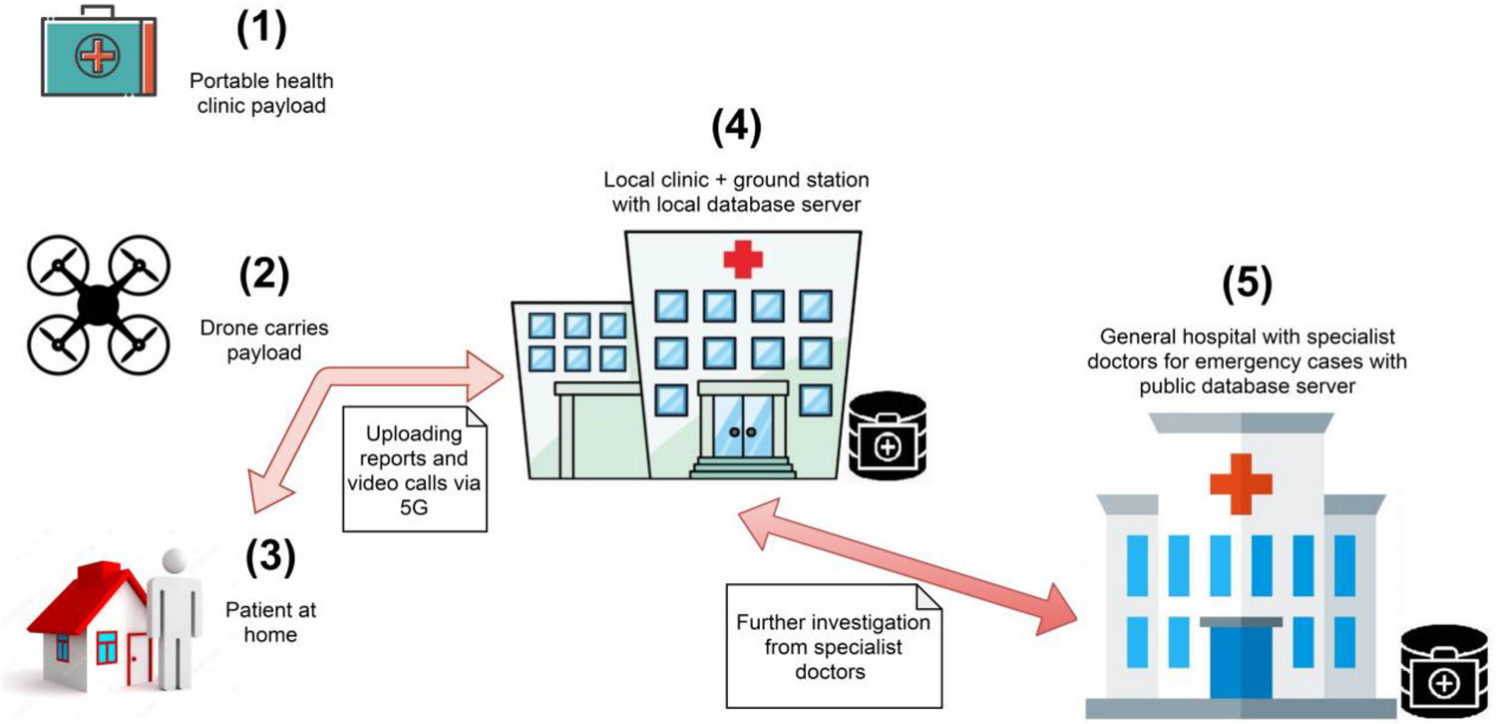
Portable health clinic (PHC) system operational procedure

A PHC system, shown in Figure 3, has been developed as an RHS for the COVID‐19 basic test during a full lockdown, where people are not allowed to visit health centres for testing, and thus there might be suspected or already confirmed COVID‐19 patients that are not aware of carrying the virus and might be spreading it to family members unintentionally. To the best of the authors' knowledge, to this day, still no scholar study the possibility of providing an automated COVID‐19 testing service to the mass at their doorsteps during curfew situations with reduced human intervention, that is, drone‐based PHC platform, and report critical conditions to local/general hospitals for further investigation/reaction. The proposed approach suggests an algorithm to categorize patients’ conditions to a multi‐level of seriousness, and also is designed to be scalable with hierarchical distribution of roles. The main five elements of the proposed approach are:

1. The PHC payload consists of the required sensors and instructions for the testing process as well as essential COVID‐19 medicine to be delivered as used when needed.
2. The multi‐UAV network acts as a portable platform and transmits the needed information to the local clinic.
3. The person/patient is at home during a full curfew to be tested.
4. The local clinic with general practitioners/doctors and local medical database is also considered to be the BS for the drone swarm with a drone operator.
5. The general hospital in the city is the best‐equipped place for COVID‐19 containment, that is, specialist doctors, medicine, infection control, treatment, and quarantine.

At the BS, the drone swarm, consisting of one LD and many SDs, is equipped with required testing sensors, then self‐configuration is conducted. After take‐off, the drones fly horizontally with a given maximum altitude to the destination area and land at the designated spot. Afterward, one approaches a SD and follows certain steps for self‐testing given by the UAV and leaves the drone‐landing zone, then the triage system classifies the case and if it is infected then the local clinic is informed for further investigation where the general hospital assistance might be needed, and the patient may be advised to take some medicine off the drone. When the swarm is done with one group of houses, it moves to another and the same procedure is repeated till the whole area is scanned then it flies back to the BS.

### 4.1 The roles and responsibilities of the proposed PHC system units

The roles and responsibilities of the proposed PHC system units are distributed hierarchically for the ease of management and scalability and are described below:

#### 4.1.1 The local clinic roles

The BS is located within the local clinic, nearby the neighbourhood to be scanned, and has the drone swarm(s) as a PHC platform fully equipped with the required payload, that is, sensors and drugs for basic COVID‐19 testing and mitigation. Also, there is a drone network operator that initiates the drones with essential mission data such as Global Positioning System (GPS) coordinates, flying speed and altitude, swarm size etc. through a dedicated server, and should always have the ability to intervene in the autonomous UAV system and manually give orders to drones as if required, for example, abort the mission. Additionally, the local clinic has a medical staff and local patients’ database, and it keeps getting periodic information about the swarm status through the LD, and if it receives a report of an infected patient, the health worker retrieves the patient's database and studies the case and either make a video call with the patient or escalate the case to the general hospital if it is urgent, contact tracing is also carried to identify potentially infected contacts. After completing the mission, the drones are disinfected and prepared again for future tours. In case of a drone failure, the BS will be reported with drone ID, last GPS coordinates, time of loss etc. to respond accordingly.

#### 4.1.2 The slave drone roles

**FIGURE 4:**
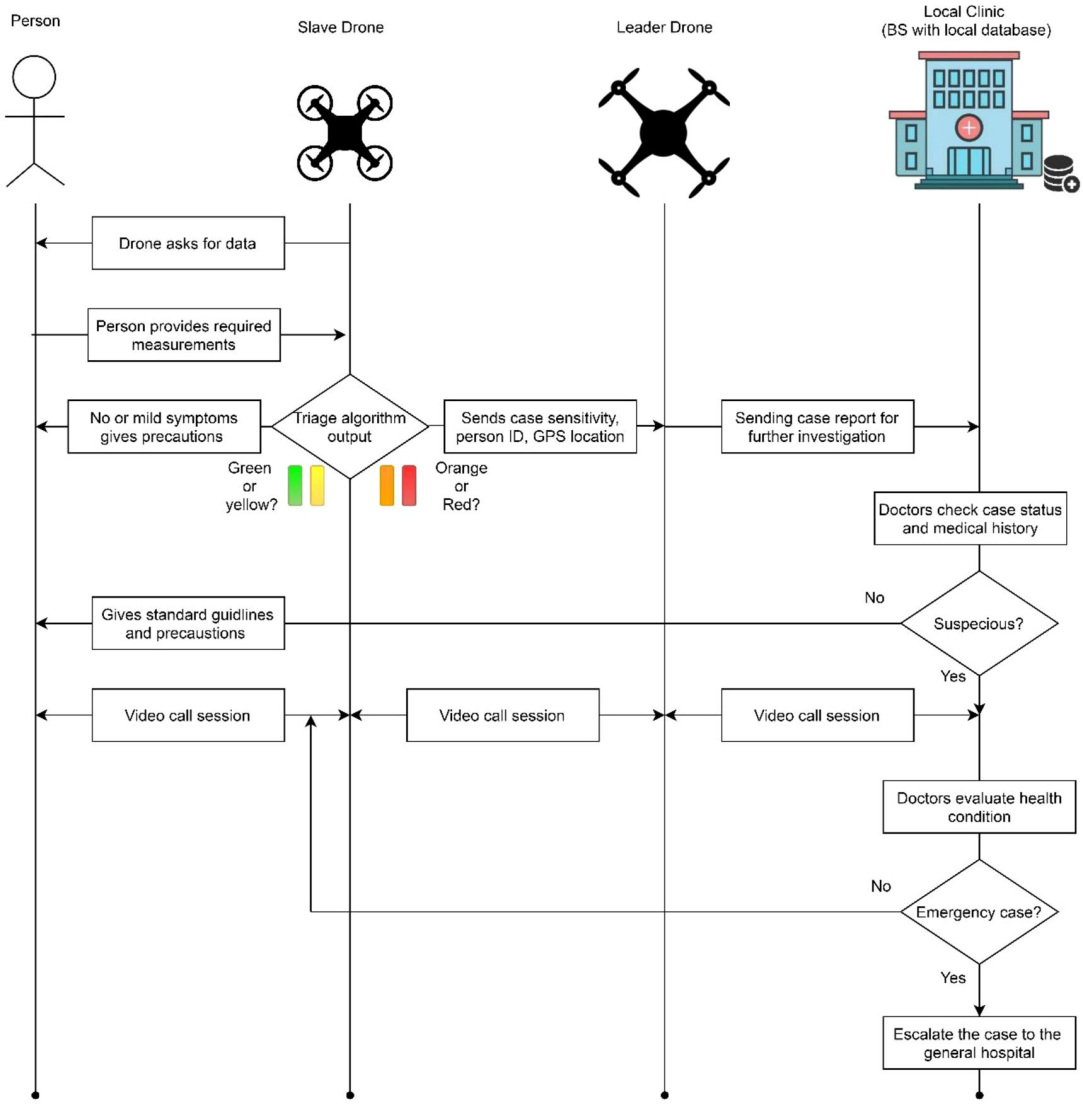
Primary screening and triaging process for COVID‐19

The SDs generally have identical payloads and mission procedures. When a SD is selected, it updates certain parameters through the self‐configuration phase including; network configuration, authentication with the LD, swarm member ID for swarm positioning, and future communications. When the swarm is ready to fly, a SD follows the LD as a reference to position itself within the swarm according to its ID to avoid colliding with other drones. When arriving, it lands according to the assigned coordinates and enters the power‐saving mode by switching off unneeded functions, for example, turning off propellers. A SD provides the LD with status reports periodically. It informs the householder of its presence and senses the person's approach through a mounted motion detection sensor. Then it gives guidance instructions for getting the required information by measuring the vital signs of a person, including the body temperature, heart rate, oxygen saturation of the blood (SpO_2_), blood pressure, and respiratory rate, through recording a video for his/her face and processing the images onboard as in [25], a detailed primary screening process is shown in Figure 4.

A SD applies a COVID‐19 triage process (CT‐process) to the measured data to classify the case severity into four categories, as mentioned in Table 4, which are; healthy or green, suspected or yellow, confirmed or orange, and emergent or red. If the case is marked with green or yellow, then only precaution instructions are given by the SD through the loudspeaker. However, if it is labelled orange or red then it might need a doctor's consultation, so a case report is sent to the LD, which in turn sends it to the BS. Then if needed, a live video call is established between a local clinic doctor and the patient through the LD and SD. After testing, the person's ID is scanned, for example, passport or national ID. After testing one person, a test results report is sent to the LD if it is positive. If the case requires more attention, a video call is placed between the patient and a remote doctor and is handled through the SD and LD. Then the same procedure is repeated for other family members and when all are tested, the slave drone puts itself in sleep mode, waiting for further instructions from the LD.

**TABLE 4:** COVID‐19 triage process (CT‐process)

No.	Symptoms	Healthy/Green (no action)	Suspicious/Yellow (no action)	Infected/Orange (consultation)	Emergent/Red (emergency)
1	Body temp. (°C)	<37.5	≥37.5	≥37.5	≥37.5
2	SpO2 (%)	≥96	≤95	≤95	≤95
3	Heart rate (bpm)	≤76	>76 and <84	≥85 and <90	≥90
4	S. blood pressure (mmHg)	≥71 and ≤81	≥81 and ≤86	≥86 and ≤91	≥91
	D. blood pressure (mmHg)	≥112 and ≤132	≥132 and ≤142	≥142 and ≤152	≥152
5	Respiratory rate (bpm)	≥12 and ≤20	≥20 and ≤22	≥22 and ≤27	≥27

#### 4.1.3 The leader drone roles

The LD first gets the required network configuration and mission information file from a dedicated server in the BS during the self‐configuration process, then it authenticates the SDs to create a drone swarm. Also, it obtains an area map of GPS coordinates. Then the LD starts taking off leaving the BS followed by the SDs, and broadcasts periodic messages of its position so other SDs can calculate and position themselves within the flying swarm, and when arriving it chooses the coordinates of the centre of the first street as a destination and assigns relevant GPS coordinates for each SD to land at; technically each slave drone is responsible for testing individuals of one house per sub‐task.

Now, all drones are landed and the LD has almost an equal number of SDs on each side creating the least distance between itself and the furthest SD for better communication, it enters the power‐saving mode by turning off unnecessary functions, that is, turning off propellers, then collects the status reports from SDs periodically and sends a periodic swarm status report to the BS. After all, SDs are done, the LD takes off to the next group of houses to be scanned using pre‐given GPS information followed by SDs, and the same process of deploying and testing starts again. Later, when the whole target area is scanned, the drone swarm returns to the BS (drone homing).

#### 4.1.4 The person being tested roles

During a full curfew, the people of the area to be scanned are already being informed by the media about the drone swarm visit for COVID‐19 testing, so residents are expecting such a visit. When one is informed by a SD, he/she goes out on the street and approaches the SD and gets familiar with the testing procedure. Afterward, the person provides his/her vital signs and scans a document to prove the identity and leaves the drone landing area, and calls for another family member for testing if any. If the person is classified as infected or emergent (orange or red labels) then a live video call from a remote doctor is expected.

#### 4.1.5 The general hospital roles

The general hospital is considered the top‐level in this approach and can supervise multiple local clinics at once. As the last resort, it should have the best available specialist doctors and medical equipment for COVID‐19 treatment and containment. When an infected case cannot be handled by a local clinic it is transferred to the general hospital for further verification. The decision is made by specialist doctors based on the patient's database, current case, and available solutions to handle the case, for example, advising the patient to be quarantined at home via a video call or sending an ambulance in case of real emergency for the treatment and quarantine at the hospital. The general hospital also performs contact tracing with keeping the privacy of the patient.

From the aforementioned roles, two operational modes can be concluded for the drone swarm, namely; the dynamic (flying) mode and the static (fixed) mode. Where the dynamic mode is when there is high network mobility during flying. On the contrary, the static mode is when the drones are landed with no mobility and entered the power‐saving mode to gather required data.

### 4.2 The proposed system states and messages

**FIGURE 5:**
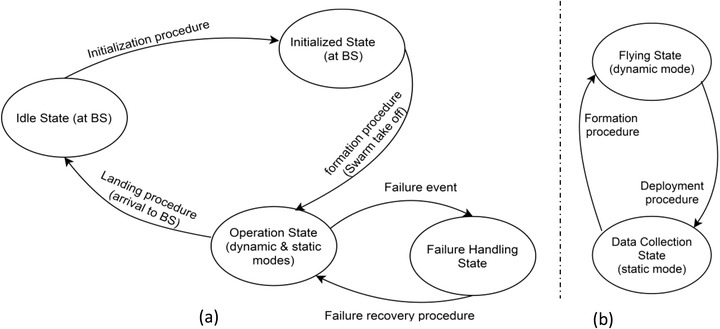
Main system state diagrams: (a) System overview (b) Operational modes

**FIGURE 6:**
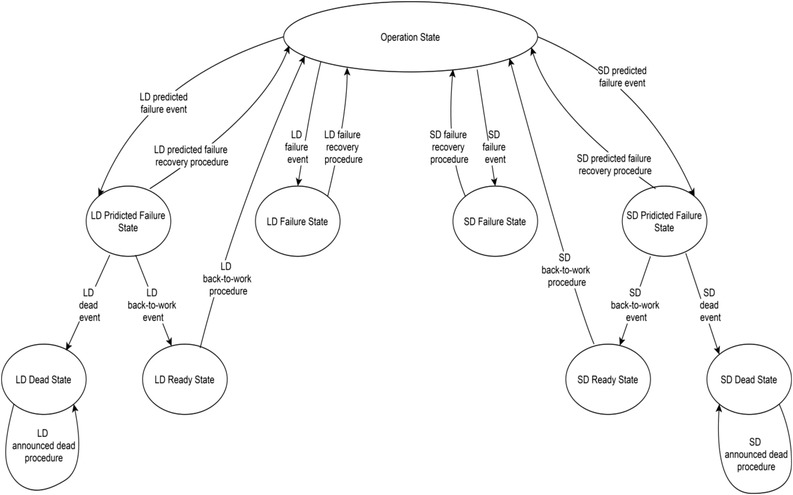
Failure handling diagram

To conduct model checking, explained in [26], the system was fully described as state diagrams. Figure 5 shows the main system diagrams and Figure 6 depicts the failure handling diagram.

There are 18 new messages in total that are used by the proposed system are shown in Table 5.

**TABLE 5:** The proposed system messages

No.	Command name	Command contents						Comm. size (Byte)
1	swarmReady	type	id_UAV_					2
2	launch	type						1
3	moveToWaypoint	type	formation type	distance	z_LD_	(x,y)_LD_		13
4	land	type	id_SD_	(x,y)_SD_				10
5	statusReport_SD_	type	id_SD_	(x,y)_SD_	battery level	temperature	available	13
6	statusReport_LD_	type	id_LD_	(x,y)_LD_	battery level	temperature	swarm status	12+12×(n)
7	caseReport	type	id_SD_	(x,y)_SD_	case severity	Scanned ID		500
8	ack	type	id_UAV_					2
9	failReport_LD_	type	id_LD_	z_LD_	(x,y)_LD_			12
10	connectionReq	type	id_SD_					2
11	preFailReport_LD_	type	id_LD_	z_LD_	(x,y)_LD_			12
12	polling	type						1
13	pollReport	type	id_UAV_	(x,y)	battery level	temperature		12
14	backReport	type	id_UAV_	(x,y)	battery level	temperature		12
15	deadReport	type	id_dead_UAV_	z_dead_UAV_	(x,y)_dead_UAV_			12
16	failReport_SD_	type	id_SD_	z_SD_	(x,y)_SD_			12
17	compensationReq	type	id_SD_	(x,y)_SD_				10
18	preFailReport_SD_	type	id_SD_	z_SD_	(x,y)_SD_			12

### 4.3 The system performance evaluation and payload effect on durability

From a technical point of view, Table 6 shows the used protocols in the proposed system.

**TABLE 6:** Networking protocols adopted by the system

Protocol	Abbreviation	Description
Dynamic Host Configuration Protocol version 6	DHCPv6	Automatically provides a host with network configurations, for example, the IP address, subnet mask and default gateway
User Datagram Protocol	UDP	Transport layer protocol
Internet Protocol version 6	IPv6	Network layer protocol
Institute of Electrical and Electronics Engineers 802.11a	IEEE 802.11a	Transmitting data over a wireless network
the Fifth Generation of cellular networks	5G	Transmitting data over broadband cellular networks
802.11e Enhanced distributed Channel Access	802.11e EDCA	Supports QoS and uses block acknowledgement to reduce traffic

#### 4.3.1 The system performance evaluation using OPNET

A simulation model is built using the (OPNET IT GURU academic edition 14.5) Network Simulation package. The goal of building this model is to locate the drone swarm, WiMax BS, and local clinic, and also generate traffic patterns as close as possible to real situations. The following assumptions are adopted when building the simulation model:

1. To simplify our simulation model, the normal SDs are assumed to be identical, while the LD and the backup LD are superior in terms of having the cellular communication capability and a more durable battery. Also, the communication circumstances are assumed to be ideal.
2. Different scenarios are simulated using OPNET with parameters specified in Table 7.
3. The traffic profiles used in simulated scenarios are given in Table 8.

**TABLE 7:** The initial settings of the simulation model

Simulation parameter	Value
Simulation time	15 min
Number of drones	1 to 100 (no video call) 1 to 14 (video call)
Network span area	2 km × 2 km
Distance between drones and formation	12 m with the linear formation
Distance between swarm and WiMax BS	1 km
Distance between swarm DMC and WiMax BS	1 km
4G adopted technique settings	WiMax technology Modulation and coding: 64‐QAM 3/4 Scheduling type: rtPS Max. sustained traffic rate: 10 Mbps Min. reserved traffic rate: 5 Mbps
WLAN adopted technique settings	802.11a (OFDM) Data rate: (6,18, 36, 54) Mbps Node buffer size = 1 M bit packet processing rate = (5000,10,000,20,000) pkt/s Block ACK: EDCA (802.11e) disabled/enabled WLAN MTU = WiMAX MTU = 1500 byte
Swarm status	Landed, power‐saving enabled and gathering data

**TABLE 8:** Traffic profiles of the simulated drone swarm

Traffic profile	Application	Description	
1	SD reporting status	(SD→LD: statusReport_SD_) Packet length = 13 byte Packet rate = 0.1 packet/s	(LD→SD: ACK) Packet length = 2 byte Packet rate = 0.1 packet/s
	LD reporting status	(LD→DMC: statusReport_LD_) Packet length = 12×(**n×12)byte Packet rate = 0.033 packet/s	(DMC→LD: ACK) Packet length = 2 byte Packet rate = 0.033 packet/s
2	SD reporting status	(SD→LD: statusReport_SD_) Packet length = 13 byte Packet rate = 0.1 packet/s	(LD→SD: ACK) Packet length = 2 byte Packet rate = 0.1 packet/s
	LD reporting status	(LD→DMC: statusReport_LD_) Packet length = 12*(n*12) byte Packet rate = 0.033 packet/s	(DMC→LD: ACK) Packet length = 2 byte Packet rate = 0.033 packet/s
	Case reporting	(SD→LD→DMC: caseReport) Packet length = 500 byte Packet rate = event‐driven	(DMC→LD→SD: ACK) Packet length = 2 byte Packet rate = event‐driven
	Video call	(SD ←→LD←→ DMC: video conference) available resolutions: Bandwidth requirements = 2 Mbit/s, 4 Mbit/s, 6 Mbit/s Frame rate = 30 frame/s	

The drone swarm is assumed to be already deployed and ready for data gathering. There are mainly two traffic profiles in normal operation mode; background traffic as periodic status reports, and video call traffic, added to the previous traffic when medical consultation is needed. See Table 8.

The two main performance parameters to be measured are the throughput and latency of the network. The term throughput refers to the user data that are meant to be transferred or exchanged and is used as a metric to evaluate the actual network performance. Additionally, Latency is the average time it takes an application on a source node to generate a packet until it is received by the application layer of the destination node. It includes delays that arise as a result of propagation, queuing and retransmission at the MAC layer. The main experimental parameters to be changed to evaluate system performance in the simulation are traffic profile, WLAN data rate, number of drones, and video size.

**FIGURE 7:**
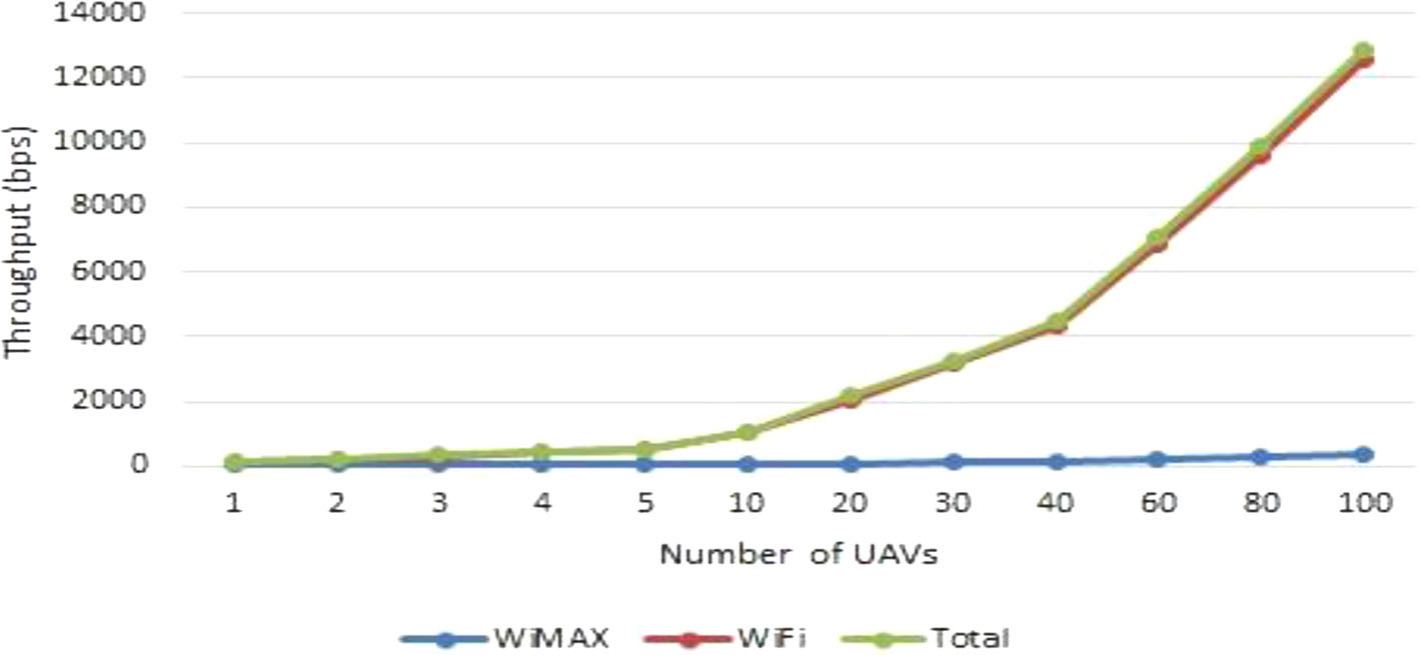
WLAN, WiMAX, and total throughput with different numbers of UAVs when there is no video call

In Figure 7, the throughput of WiMAX, WLAN, and the total throughput is measured when there is no video call with a different number of UAVs, here only traffic profile 1 is taking place, and it shows that the total throughput and traffic exchanging is mostly done in the WLAN as intra‐swarm traffic by periodically sending status reports from each SD to the LD (every 10 s), while the WiMAX throughput is much less as it only includes the LD sharing the swarm status report with the BS periodically (every 30 s).

**FIGURE 8:**
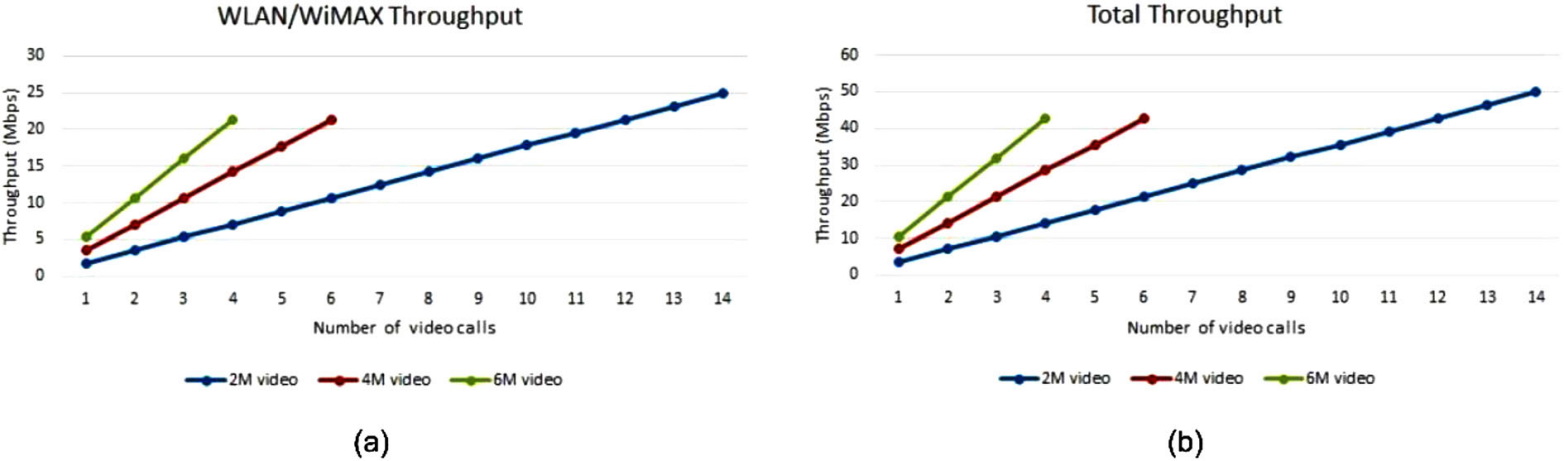
Throughput with a different number of video call instances: (a) WiMax/WLAN throughput (b) Total throughput

When a video call is in place at a WLAN data rate of 54 Mbps, that is, running traffic profile 2, throughput is expected to be significantly higher than exchanging only status reports as more data is being transferred. Therefore, unlike the result shown in Figure 7, where WLAN and WiMAX throughput vary a lot, it is expected that WLAN and WiMAX have almost the same throughput as shown in Figure 8. It is worth mentioning that a limited number of 14, 6, and 4 instant video calls of 2, 4, and 6 Mbit can be supported respectively.

**FIGURE 9:**
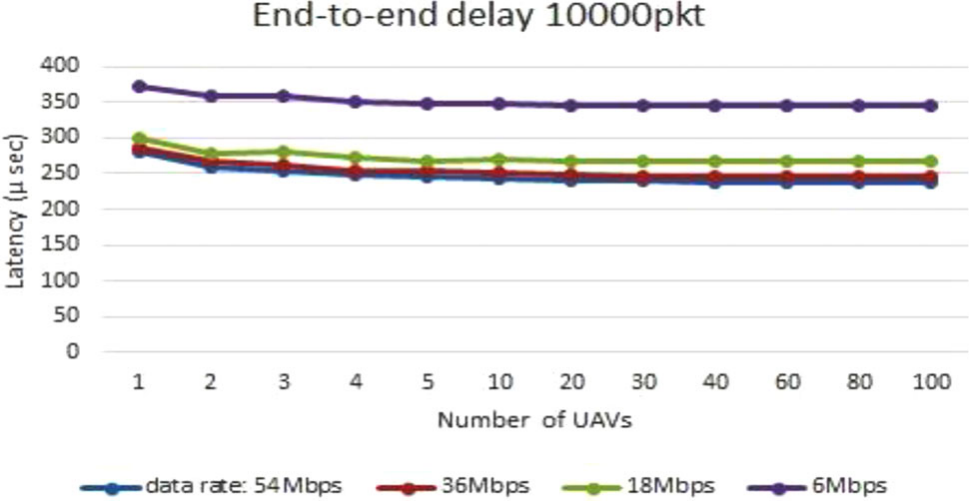
No video call latency with different data rates, and packet processing rate of 10,000 pkt/s

**FIGURE 10:**
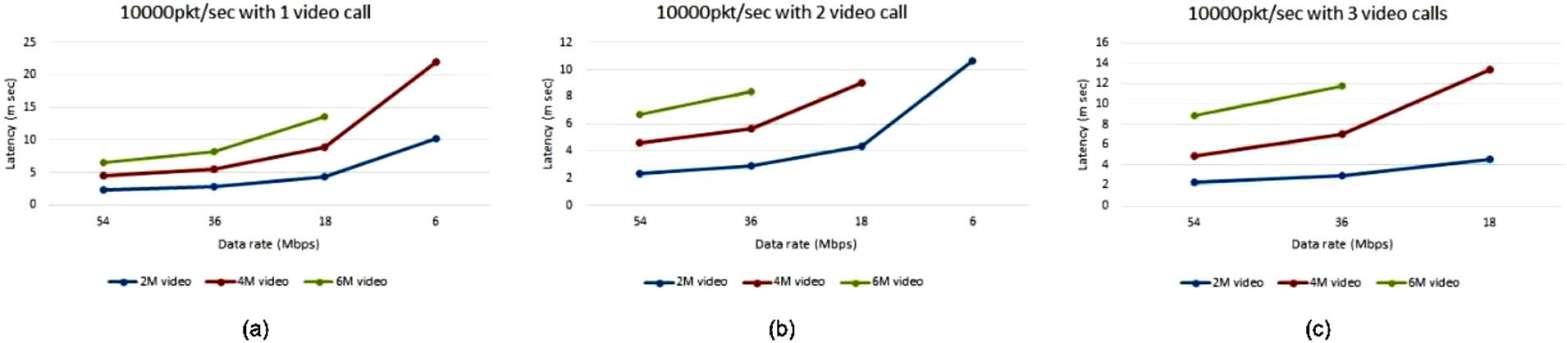
Video call latency at different data rates with a video bandwidth of (a) 2 Mbit (b) 4 Mbit (c) 6 bit

When only traffic profile 1 is activated the periodic status reports are exchanged with no video call, and the latency of different data rates is around 250–350 μs for 10,000 packet/s packet processing rate as shown in Figure 9, as there is not much traffic included. However, a latency range of around (3–10 m), (5–22 m), and (6–14 m) seconds are calculated during one (2, 4, and 6 Mbit of bandwidth) video call instances respectively with different data rates and a packet processing rate of 10,000 packet/s as shown in Figure 10a, while Figure 10b,c also shows the latency but for 2 and 3 simultaneous video calls respectively.

**FIGURE 11:**
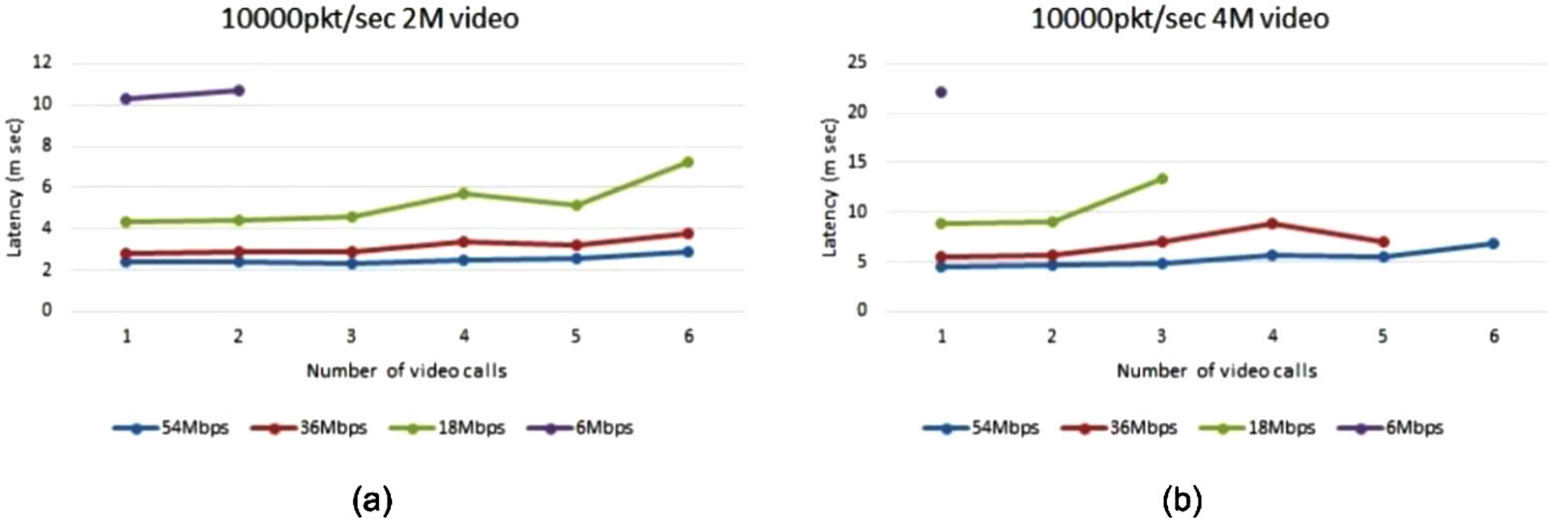
The effect of data rate on latency when running a different number of video calls with different resolutions: (a) 2 Mbit video (b) 4 Mbit video

The lower the data rate, the more time it takes for packets to travel through the network and hence higher latency, as shown in Figure 11.

**FIGURE 12:**
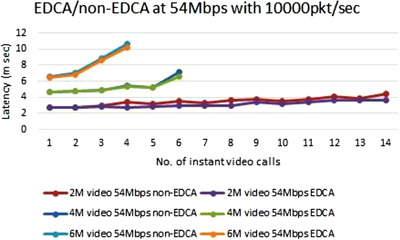
EDCA effect on latency

Network latency can be further reduced by applying the principle of block acknowledgment, which is part of 802.11e Enhanced Distributed Channel Access (EDCA), where a maximum of 64 frames are treated as a single block and only one block acknowledgment is sent as shown in Figure 12.

#### 4.3.2 The effect of payload on durability

**FIGURE 13:**
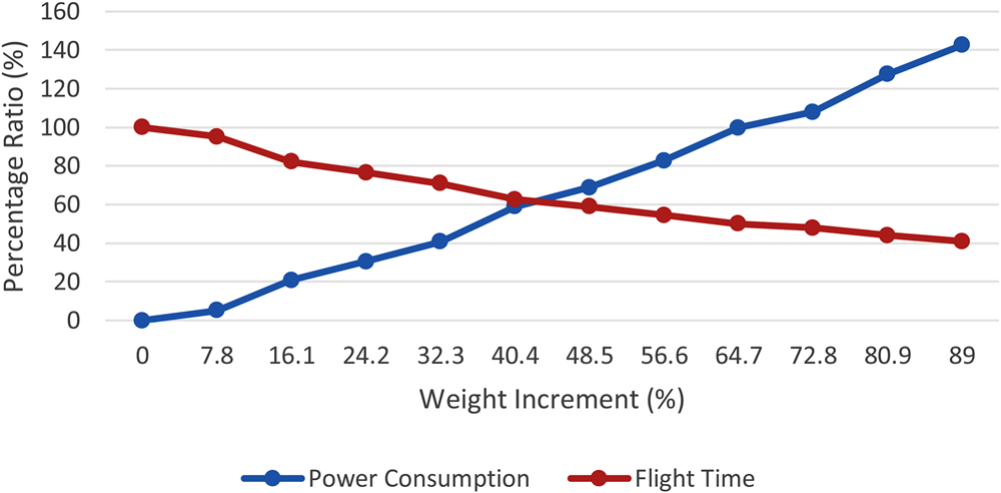
Drone's payload relation with power consumption and flight time

Measuring power consumption in research are considered [27, 28, 29, 30, 31, 32]. According to Table 3, the needed payload adds an additional weight to the drone, thus, this will have a negative impact on its flight time. As stated in [33, 34], the drone payload and its effect on power consumption as well as flight time is measured, the following relations can be concluded as shown in Figure 13.

## 5 DISCUSSION AND COMPARISON WITH OTHER WORKS

During public health emergencies like the COVID‐19 pandemic, deploying the PHC and other related RHS technologies in hotspot regions is helpful in terms of minimizing the risk of the virus transmission to frontline healthcare professionals and reducing their stress. Also, to avoid crowded places where there is no need for people to come in person to clinics/hospitals. Despite having so many advantages, UAV‐based RHS poses challenges including [5, 35, 36]:

1. Privacy concerns: The gathered data should not be used for improper purposes.
2. Regulatory issues: Each flight should be licensed by authorized bodies.
3. Suboptimal performance: Especially when drones are fully automated.
4. Meeting performance and QoS requirements.
5. Limited energy supply: Solar‐based charging system is needed to alleviate this issue.
6. Appropriate financial and human resources.
7. Sensitization of communities and stakeholders before and during PHC implementation.
8. Drone integration into the health supply chain: Should consider evaluating drone acceptability, profitability, and performance. Table 9 shows a comparative study.

**TABLE 9:** A comparative analysis of the proposed approach with existing drone‐based systems

Authors	Year	System type	**Features								
			A	B	C	D	E	F	G	H	I
Mohammed et al. [37]	2020	IoT‐based drone system for COVID‐19 detection	✓	**×**	**×**	**×**	✓	**×**	✓	**×**	**×**
Manigandan et al. [38]	2020	Drone detection of COVID‐19 with no human interventions	✓	**×**	**×**	**×**	**×**	✓	**×**	**×**	**×**
Soni et al. [39]	2020	UAS for consumer utilities in COVID‐19 pandemic	**×**	**×**	**×**	**×**	**×**	**×**	**×**	✓	✓
Elbir et al. [40]	2020	Vehicular Network for combating the spread of COVID‐19	**×**	**×**	**×**	**×**	**×**	**×**	✓	**×**	✓
Sharma et al. [5]	2021	Drone delivery dynamic models in COVID‐19 hotspots	✓	**×**	**×**	**×**	**×**	✓	**×**	✓	**×**
Patchou et al. [41]	2021	Drone‐based efficient parcel delivery during COVID‐19	**×**	**×**	**×**	**×**	**×**	**×**	**×**	✓	✓
Alsamhi et al. [42]	2021	Blockchain for multi‐drone to combat COVID‐19	✓	**×**	**×**	**×**	**×**	✓	✓	✓	**×**
Kumar et al. [35]	2021	Drone‐based network for COVID‐19 operations	✓	**×**	✓	✓	✓	✓	✓	**×**	**×**
Proposed work	2021	Drone swarm as a PHC in COVID‐19 hotspots	✓	✓	✓	✓	✓	**×**	**×**	✓	**×**

**Features: A: Covid‐19‐related data collection, B: Multi‐level classification, C: Announcement, D: Person identification, E: Real‐time video communication, F: Sanitization, G: Surveillance, H: Delivery, I: Communication relay.

## 6 CONCLUSIONS

PHC systems offer an inexpensive, usable set of portable sensors to transfer clinical data to a remote doctor to make an accurate decision. This paper mentioned relevant existing and future public health implications arising from the coronavirus spread. It presented an overview of how some drone‐based initiatives have been developed to handle the situation. Our proposed PHC platform and its related methods is a leading contributor to public health responses, particularly for residents in prolonged curfews, and suggests a strong possibility of positive impact. In this paper, we redesigned the current PHC platform as a means to contain the COVID‐19 spread, as well as suggested a COVID‐19 triage process (CT‐process), that classifies the patients on whether they need to be connected on a video call to a doctor and moved to a clinic for further inspection and treatment, by considering and analysing the main symptoms of COVID‐19, such as body temperature, heartbeat, respiratory rate, blood pressure, and SpO2. Additionally, the proposed approach allows for the delivery of essential coronavirus mitigation drugs. By achieving this, it minimizes the COVID‐19 transmission risk and reduces psychological stress on frontline medical personnel, and maximizes the availability of healthcare resources to be used by patients who are most in desperate need of them.

## FUNDING INFORMATION

The authors received no funding for this work.

## CONFLICT OF INTEREST

The authors declare that they have no relevant or material financial interests that relate to the research described in this paper.

## AUTHOR CONTRIBUTIONS

M.S.Q.: Conceptualization; investigation; methodology; resources; validation; writing—original draft; writing—review and editing. Q.I.A.: Resources; supervision; validation

## Data Availability

Data sharing is not applicable to this article as no new data were created or analysed in this study.
